# Excitability of Aβ sensory neurons is altered in an animal model of peripheral neuropathy

**DOI:** 10.1186/1471-2202-13-15

**Published:** 2012-01-30

**Authors:** Yong Fang Zhu, James L Henry

**Affiliations:** 1 Farncombe Family Digestive Health Research Institute, McMaster University, 1280 Main St. West, HSC 3N5C, Hamilton, Ontario. Canada; 2 Department of Psychiatry and Behavioural Neurosciences, McMaster University, 1280 Main St. West, HSC 4N35, Hamilton, Ontario, Canada; 3 Michael DeGroote Institute for Pain Research and Care, and Department of Psychiatry and Behavioural Neurosciences, McMaster University, Hamilton, ON, Canada

**Keywords:** Neuropathic pain, Primary afferent neuron, Hyperexcitability, Ectopic discharge, Muscle spindle neuron, Dorsal root ganglion

## Abstract

**Background:**

Causes of neuropathic pain following nerve injury remain unclear, limiting the development of mechanism-based therapeutic approaches. Animal models have provided some directions, but little is known about the specific sensory neurons that undergo changes in such a way as to induce and maintain activation of sensory pain pathways. Our previous studies implicated changes in the Aβ, normally non-nociceptive neurons in activating spinal nociceptive neurons in a cuff-induced animal model of neuropathic pain and the present study was directed specifically at determining any change in excitability of these neurons. Thus, the present study aimed at recording intracellularly from Aβ-fiber dorsal root ganglion (DRG) neurons and determining excitability of the peripheral receptive field, of the cell body and of the dorsal roots.

**Methods:**

A peripheral neuropathy was induced in Sprague Dawley rats by inserting two thin polyethylene cuffs around the right sciatic nerve. All animals were confirmed to exhibit tactile hypersensitivity to von Frey filaments three weeks later, before the acute electrophysiological experiments. Under stable intracellular recording conditions neurons were classified functionally on the basis of their response to natural activation of their peripheral receptive field. In addition, conduction velocity of the dorsal roots, configuration of the action potential and rate of adaptation to stimulation were also criteria for classification. Excitability was measured as the threshold to activation of the peripheral receptive field, the response to intracellular injection of depolarizing current into the soma and the response to electrical stimulation of the dorsal roots.

**Results:**

In control animals mechanical thresholds of all neurons were within normal ranges. Aβ DRG neurons in neuropathic rats demonstrated a mean mechanical threshold to receptive field stimulation that were significantly lower than in control rats, a prolonged discharge following this stimulation, a decreased activation threshold and a greater response to depolarizing current injection into the soma, as well as a longer refractory interval and delayed response to paired pulse electrical stimulation of the dorsal roots.

**Conclusions:**

The present study has demonstrated changes in functionally classified Aβ low threshold and high threshold DRG neurons in a nerve intact animal model of peripheral neuropathy that demonstrates nociceptive responses to normally innocuous cutaneous stimuli, much the same as is observed in humans with neuropathic pain. We demonstrate further that the peripheral receptive fields of these neurons are more excitable, as are the somata. However, the dorsal roots exhibit a decrease in excitability. Thus, if these neurons participate in neuropathic pain this differential change in excitability may have implications in the peripheral drive that induces central sensitization, at least in animal models of peripheral neuropathic pain, and Aβ sensory neurons may thus contribute to allodynia and spontaneous pain following peripheral nerve injury in humans.

## Background

Neuropathic pain is associated with exaggerated responses to painful stimuli (hyperalgesia), pain provoked by normally innocuous stimulation (allodynia), abnormal spontaneous sensations (dysesthesia) and a spontaneous burning pain [1-4]. This type of chronic pain remains generally undertreated, perhaps at least partially due to a lack of mechanism-based treatments. Currently, the mechanisms of neuropathic pain following peripheral nerve injury remain unresolved. The physiological consequences of peripheral nerve damage associated with neuropathic pain readouts in animal models have provided detailed information suggesting an involvement of C-fiber sensory neurons in mediating the functional changes in these models [5,6]. This is consistent with the classic concept that pain and central sensitization are largely due to sensory input from C-fiber afferents.

However, compelling evidence from animal models suggests a role of large A-fiber, heavily myelinated neurons in mediating neuropathic pain. For example, intracellular recordings *in vitro *from dorsal root ganglia (DRG) in an axotomy model, where the L5 spinal nerve was cut, showed enhanced responses of A-type neurons to intracellular injection of depolarizing current [7] and exhibited spontaneous discharge, which was not observed in C neurons [8]

Spontaneous activity and increased excitability have also been demonstrated in A-type neurons in vitro in the dorsal root ganglion (DRG) compression model [9,10] and in the chronic constriction injury model [11,12] of neuropathic pain, although this does not seem to be restricted only to A-type neurons [9,13]. Classification of neurons in *in vitro *studies can be only on the basis of conduction velocity or soma size, but classification cannot be based on functional criteria. Full functional classification is available in *in vivo *studies.

In axotomy models *in vivo *extracellular recordings from sensory nerve fibers [14,15] and from DRG neurons [16,17] have demonstrated an increase in excitability of A-fiber neurons. However, in such studies, where peripheral nerves are cut, functional classification of sensory neurons is also inaccessible.

Most types of neuropathic pain do not arise from cut nerves but from physically or metabolically induced neurotrauma. There have been some *in vivo *studies based on nerve intact models of peripheral neuropathic pain. Extracellular recordings from dorsal root fibers in the CCI model have shown a general increase in excitability of all neuron types [18]. Khan et al. [19] have reported, on the other hand, that in the streptozotocin model of diabetic neuropathy single Aδ-fiber and Aβ-fiber sensory axons in the tibial nerve develop ectopic discharge and higher spontaneous activity, and exhibit lower activation threshold to mechanical stimuli. The changes in sensitivity and in axon excitability were unaltered by treatment with i.p. resiniferatoxin to deplete C-fiber neurons.

We have also been investigating mechanisms of nociceptive pain in an intact nerve animal model of peripheral neuropathy, in our case by inserting a polyethylene cuff around one sciatic nerve [20]. Our previous studies demonstrated that myelinated afferents mediate the increased responses of spinal dorsal horn nociceptive neurons to noxious [21] and innocuous stimulation [22] and that the peripheral drive exciting these neurons is due to ectopic discharge arising from multiple sites along the sensory nerve [23,24]

Little is known, however, regarding the specific functional type of myelinated neuron undergoing changes in excitability. For example, the studies on the CCI [18], the steptozotocin [19] and the DRG compression [9] models did not classify neurons other than on the basis of conduction velocity. Further, the studies on the DRG compression model did not differentiate between different types of A neuron. Studies on axotomy models lose the ability to classify neurons functionally. Tal et al., [25] reported that spontaneous discharge and increased mechanosensitivity varies depending on the type of peripheral tissues innervated.

In view of the contradictory evidence regarding the specific neuron types undergoing the changes in excitability in *in vivo *electrophysiological studies on nerve intact models of neuropathic pain, it was considered important to understand the specific population of Aβ-fiber DRG neurons possibly implicated in mechanisms of neuropathic pain. Clear classification criteria are available in the literature based on responses to natural stimulation [26]. Although earlier *in vivo *studies on sensory neurons in neuropathic pain models had access to stimulation of peripheral receptive fields this was not done in classification of neuron types; rather, classification was done on the basis of conduction velocity. As a result, we set out to record in vivo from single Aβ-fiber DRG neurons that were functionally classified on the basis of their responses to natural stimulation of peripheral fields, comparing excitability of neurons in cuff-implanted model animals with excitability of neurons in surgically naive animals. Classification was also based on conduction velocity and other criteria defined by Fang et al. [27]. As our aim was to gain information on changes in excitability of these classified neurons, we recorded intracellularly in order to determine excitability by intracellular injection of depolarizing current, by application of von Frey filaments to the peripheral receptive field and by electrical stimulation of dorsal roots.

## Results

### Withdrawal response in the behavioral von Frey test

**Figure 1:**
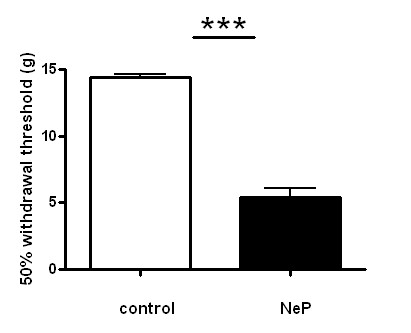
**Comparison of 50% withdrawal threshold between control and neuropathic rats**. Withdrawal threshold to mechanical stiulation of the plantar surface of the ipsilateral hind paw with von Frey filaments, recorded on the same day immediately before the acute electrophysiological experiment, during the third week after model induction, in control (n = 60) and neuropathic (n = 64) animals.

Stimulation of the plantar surface of the hind paw with von Frey filaments evoked a withdrawal response in control animals, with hairs exerting pressures of 10-100 g. Three weeks after cuff implantation on the sciatic nerve on the right side, the rats fully developed behavioral signs of mechanical hypersensitivity on the affected hind limb, a parallel to the tactile allodynia reported by people with neuropathic pain. Neuropathic animals responded to filaments that the control animals ignored, i.e. 0.001-6.0 g, which evoked a clear withdrawal on the nerve-injured side. Furthermore, in neuropathic rats the withdrawal was exaggerated in amplitude and duration, and it was frequently accompanied by licking of the paw. Withdrawal thresholds were 14.44 ± 0.221 g in control animals (*N *= 60) and 4.52 ± 0.69 g in neuropathic animals (*N *= 64). Comparison of the data indicated a difference between these groups *P *< 0.0001. The data are shown in Figure 1.

### Excitability of the receptive field measured by responses to application of von Frey filaments

To determine whether changes in properties of peripheral receptors might contribute to the mechanical hypersensitivity that characterizes this model, von Frey filaments were applied to the peripheral receptive fields of neurons studied in the electrophysiological recordings. Calibrated von Frey filaments were applied to the identified receptive field areas as a tactile stimulation and the minimum filament that elicited an AP in the soma was recorded.

**Figure 2:**
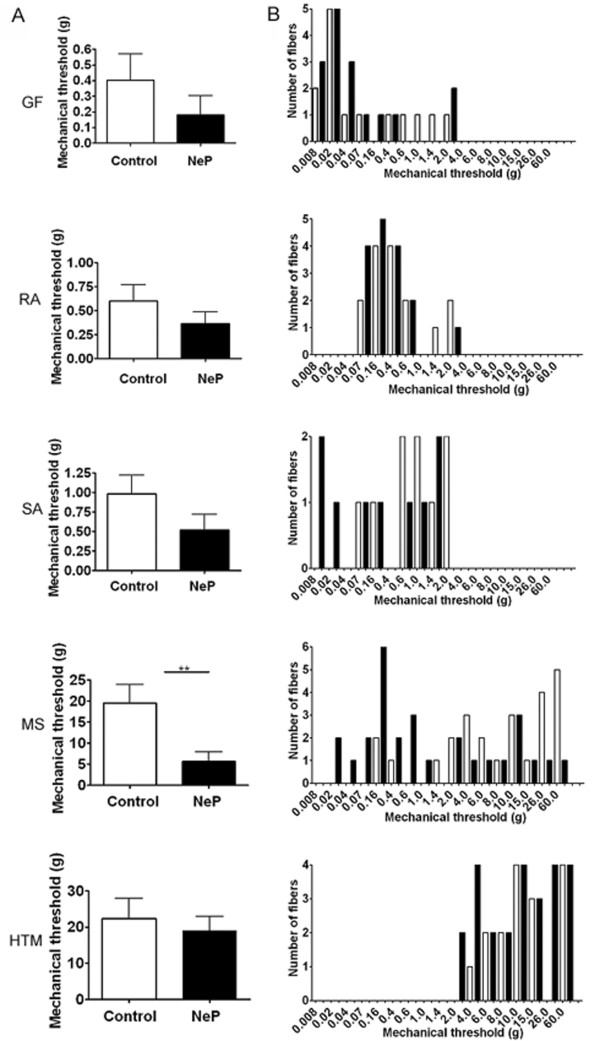
**Comparison of the mechanical response threshold of Aβ-fiber neurons to application of von Frey filaments to the peripheral receptive fields of control and neuropathic rats**. The column on the left shows the mean mechanical response thresholds of GF, RA, SA, MS and HTM neurons in control (oen bars) and neuropathic (NeP; filled bars) rats. Note that the activation threshold of MS neurons was significantly lower in neuropathic than in control animals. The column on the right shows the distribution of the mechanical activation thresholds of the individual neurons. There is a leftward shift in the distribution of the SA and MS neurons in the neuropathic animals compared with the control animals. **P < 0.01. Unpaired t-test.

The mechanical thresholds with von Frey filaments of all Aβ DRG neurons during electrophysiology recording are shown in Figure 2. The mechanical thresholds of GF (*N *= 14), RA (*N *= 13), SA (*N *= 9), MS (*N *= 25) and Aβ HTM (*N *= 16) neurons in control rats were within the range 0.008-2 g, 0.07-2 g, 0.07-2 g, 0.16-60 g and 4-60 g, respectively. In neuropathic rats, the thresholds of GF (*N *= 16), RA (*N *= 16) neurons were the same as in control animals. However, in neuropathic animals the thresholds of SA (*N *= 9), MS (*N *= 28) and HTM (*N *= 19) neurons were shifted lower, to 0.008-2 g, 0.02-2 g and 2-60 g, respectively. The mean mechanical threshold of the MS neurons was significantly lower in neuropathic rats than in control rats: 19.48 ± 4.459 g in control vs. 5.64 ± 2.311 g in neuropathic neurons (*P *= 0.0064). The other subtypes of Aβ-fiber neurons showed no significant difference. Thresholds of hair neurons were 0.40 ± 0.170 g in control and 0.18 ± 0.124 g in neuropathic neurons (*P *= 0.2919). Those in RA neurons were-0.60 ± 0.170 g vs. 0.37 ± 0.118 g in control and neuropathic animals, respectively (*P *= 0.2679). Those in SA neurons were 0.98 ± 0.237 g in control animals and 0.52 ± 0.200 g in neuropathic animals (*P *= 0.1556). Those in HTM neurons in control animals were 22.31 ± 5.674 g vs.19.08 ± 3.948 g in neuropathic animals (*P *= 0.6321).

Some neurons in neuropathic rats exhibited ongoing discharge consisting of fast excitatory APs. For example, C-fiber cool neurons usually discharge at room temperature and MS neurons can discharge when the leg is fixed in an extended position in both control and neuropathic rats. In the present study, ongoing discharge was recorded from C-fiber cool neurons and Aβ-MS neurons in both control (*N *= 62) and neuropathic rats (*N *= 70). As this study focused on differences in responses evoked by applied stimuli between control and model animals these neurons were excluded from the present analysis.

**Figure 3:**
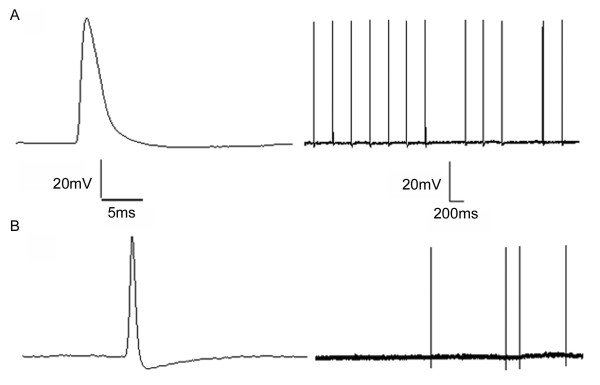
**Persisting discharge in response to stimulation of the peripheral receptive field in neuropathic rats**. This figure shows a persisting discharge seen in DRG neurons in neuropathic rats that was not observed in control rats-an Aβ-fiber HTM neuron (A), Aβ-fiber hair neuron (B). The APs on the left are the first AP in the on-going discharge shown on the right at a slower sweep speed.

In neuropathic rats, 6 of 39 Aβ HTM, 1 of 10 hair Aβ LTM neurons demonstrated certain electrical signs that are reasonably interpreted as an increase in excitability (Figure 3). An increased number of action potentials could be evoked by stimulation of the receptive field, and the discharge persisted for several minutes after removal of the stimulus from the receptive field. It is important to note that in control animals none of the 26 Aβ HTM or of the 50 Aβ LTM neurons demonstrated any prolonged discharge following application of peripheral stimuli.

### Excitability of the soma measured by responses to injection of depolarizing current

**Figure 4:**
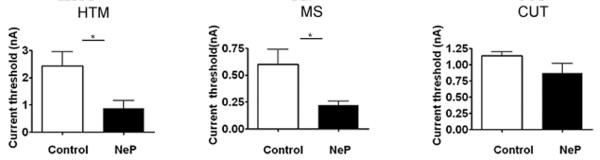
**Comparison of the activation threshold of Aβ-fiber neurons in response to intracellular current injection, between control and neuropathic (NeP) rats**. The current threshold was defined as the minimum current required to evoke an AP by intracellular current injection. Excitability of the DRG somata was significantly increased, as indicated by the decreased activation threshold in MS and HTM neurons. CUT, cutaneous neurons, include Aβ-fiber LTM GF, RA, and SA neurons. Asterisks above the graph indicate the significant difference between control and neuropathic animals: *p < 0.05. Unpaired t-test.

The AP responses to intracellular depolarizing current pulse injection were tested to determine whether there is a difference in soma excitability induced by the peripheral neuropathy. Figure 4 illustrates the threshold currents that elicited APs in control and neuropathic animals. Compared to the control group, Aβ-fiber HTM neurons and Aβ-fiber LTM MS neurons in neuropathic animals showed a significant difference from controls; activation threshold of HTM neurons was 2.430 ± 0.526 nA (*N *= 15) vs. 0.87 ± 0.284 nA (*N *= 15) in control and neuropathic rats, respectively (*P *= 0.0230). Activation threshold of MS neurons was 0.60 ± 0.138 nA in control (*N *= 17) and 0.18 ± 0.124 g in neuropathic (*N *= 17) rats (*P *= 0.0216). There was no significant difference in Aβ-fiber LTM cutaneous neurons in control vs. model animals (1.14 ± 0.062 nA, *N *= 16, in control animals vs. 0.87 ± 0.154 nA, *N *= 16, in neuropathic animals; *P *= 0.1294).

**Figure 5:**
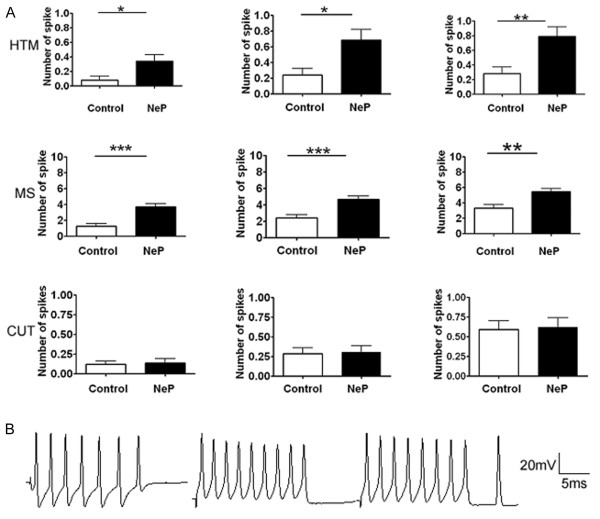
**A comparison the repetitive discharge characteristics of DRG cells produced by intracellular current injection**. The discharge was evoked by injecting a series of depolarizing current pulses into DRG soma through the recording electrode. Differences in the discharges of the 3 categories of Aβ-fiber neuron are summarized. **A**. Colum bars showing the number of APs evoked by different magnitudes of intracellular depolarizing current injection. Left, 1 nA, 20 ms. Mid, 1.5 nA, 20 ms. Right, 2 nA, 20 ms. **B**. Representative examples of raw recordings to show the greater number of APs evoked by intracellular current injection in MS neurons. APs were evoked by current pulses of 2 nA, 20 ms. Left, control rat. Mid, neuropathic rat. Right, neuropathic rat. Details of the abbreviation are as indicated in Figures 2 and 4. Asterisks above the graph indicate a significant difference between control and neuropathic animals. *p < 0.05, **P < 0.01; ***p < 0.001.

The effects of peripheral neuropathy on repetitive discharge were analyzed quantitatively by frequency-current analysis. Figure 5A shows the number of APs elicited with different current strengths, comparing control vs. neuropathic rats. Compared to the control group, Aβ-fiber HTM neurons and Aβ-fiber LTM MS neurons in the neuropathic rats showed a significant difference, while Aβ-fiber LTM cutaneous neurons showed no difference. Subthreshold oscillations and bursting patterns of discharge, which have been implicated in generating ectopic discharge in an axotomy model of neuropathic pain (Liu et al., 2000), were not observed in this intact model.

For Aβ HTM neurons, the results were based on 25 neurons in control rats and 38 neurons in neuropathic rats. With a 1.5 nA, 20 ms current injection, the number of APs in control rats was 0.08 ± 0.055 and in neuropathic rats it was 0.34 ± 0.087 (*P *= 0.0275). With a 2 nA, 20 ms current injection, the number of APs in control rats was 0.24 ± 0.087 while neuropathic rats this was 0.68 ± 0.137 (*P *= 0.0181). With a 2.5 nA, 20 ms current injection, the number of APs in control rats was 0.28 ± 0.092 while neuropathic rats it was 0.79 ± 0.132 (*P *= 0.0059).

For MS neurons, the results were based on 25 neurons in control rats and 38 neurons in neuropathic rats. With a 1.5 nA, 20 ms current injection, the number of APs in control rats was 1.28 ± 0.345 while in neuropathic rats it was 3.72 ± 0.397 (*P *< 0.0001). With a 2 nA, 20 ms current injection, the number of APs in control rats was 2.40 ± 0.378 and 4.66 ± 0.411 in neuropathic rats (*P *= 0.002). With a 2.5 nA, 20 ms current injection, the number of APs in control rats was 3.31 ± 0.448 while it was 5.44 ± 0.400 in neuropathic rats (*P *= 0.0012).

For Aβ-fiber LTM cutaneous neurons, the results were based on 66 neurons in control animals and 66 in neuropathic animals. With the 1.5 nA, 20 ms current injection, the number of spikes in control rats was 0.12 ± 0.040 and in neuropathic rats it was 0.136 ± 0.052 (*P *= 0.8193). With the 2 nA, 20 ms current injection, the number of spikes in control rats was 0.28 ± 0.070 and 0.30 ± 0.081 in neuropathic rats (*P *= 0.8880). With the 2.5 nA, 20 ms current injection, the number of APs in control rats was 0.59 ± 0.112 and neuropathic rats it was 0.62 ± 0.120 (*P *= 0.8538).

Figure 5B shows typical discharge patterns elicited in MS neurons by 2 nA current pulses with a duration 20 ms. In this figure, control animals showed 7 APs while neuropathic rat showed 9 APs with the same current pulse injection, which was the maximal number of APs observed in both groups using 2 nA current pluses.

### Excitability of the axon measured by responses to dorsal root stimulation

**Figure 6:**
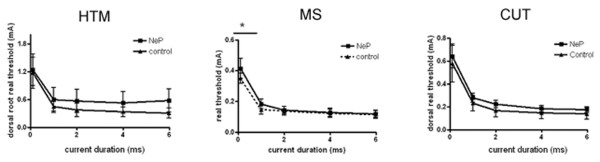
**Comparison of current activation threshold of the Aβ-fiber neurons in response to stimulation of the dorsal roots, between control and neuropathic rats**. Dorsal root current threshold was defined by the chronaxie curve (threshold-duration), which was determined as the minimum stimulus current to the dorsal root sufficient to evoke a soma AP with pulses of 0.1 ms, 1 ms, 2 ms, 4 ms and 6 ms duration. Figures show a reduction in the rheobase in DRG neurons in neuropathic animals. Details of abbreviations are as indicated in Figures 2 and 4.

The responses to axonal stimulation by delivering single current pulses of 8 mA to the dorsal root were examined. Dorsal root excitability was determined as the chronaxie curve (threshold-duration curve), which was derived by determining the minimum current applied to the dorsal root that just evoked a soma AP with pulse durations of 0.1 ms, 1 ms, 2 ms, 4 ms and 6 ms. The data are shown in Figure 6. MS neurons showed significantly lower current intensity threshold with 0.1 ms stimulation (0.35 ± 0.032 mA, N = 5; 0.46 ± 0.032 mA, N = 7; *P *= 0.041).

**Figure 7:**
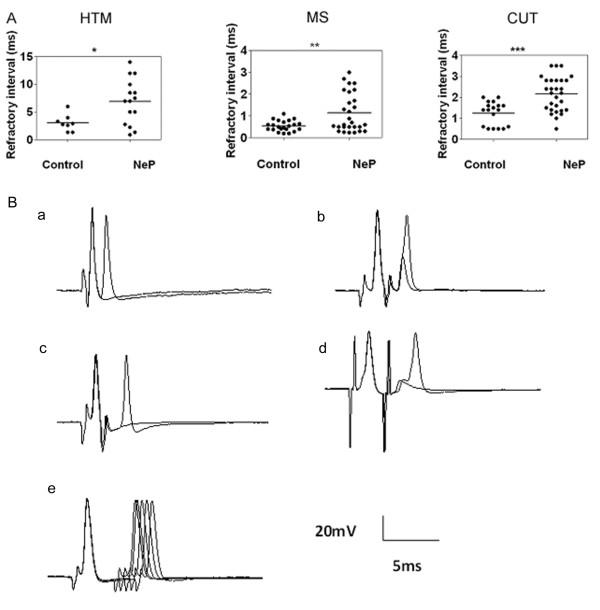
**Comparison of the repetitive discharge characteristics of DRG cells evoked by dorsal root stimulation between control and neuropathic (NeP) rats**. Dorsal root repetitive discharge characteristics were tested by measuring the refractory interval of two APs elicited by paired pulse stimulation of the dorsal roots. **A**. Scatter plots showing the distribution of the refractory interval variables with the median (horizontal line) superimposed in each case. Asterisks above the graph indicate a significant difference between control and neuropathic animals. *p < 0.05, **P < 0.01; ***p < 0.001. Abbreviations are as indicated in Figures 2 and 4. **B**. Representative examples of somatic APs evoked by paired pulse dorsal root stimulation with variable inter-stimulus intervals. (a) a MS neuron in a control rat (interval 0.3, 0.2 ms), (b) a MS neuron in a neuropathic rat (interval at 1.8, 2 ms). The data show a longer refractory interval in neuropathic rats. (c, d) MS neurons in neuropathic rats (left: interval: 1.6, 1.5 ms; right: interval: 2.5, 2.6 ms). The data show not only a longer refractory interval but also a delayed second AP response to the second stimulus. (e) a MS neuron in a neuropathic rat (interval. 5.5, 5, 4.5, 4, 3.5, 3 ms). The data show that the second AP failed at an interval of 3 ms, the second AP was delayed at 3.5 ms and the second AP was elicited when at an interval of 4 ms.

To investigate the fast process of recovery from an AP in each subtype of afferent neuron, paired-pulse stimuli were delivered to the dorsal root. The RI of Aβ neurons showed a significant difference between neuropathic and control rats. Figure 7A displays the RI distributions for individual neurons in each Aβ neuron subtype in control and neuropathic rats. In control rats, Aβ-fiber HTM neurons had the longest RI (3.05 ± 0.530 ms, *N *= 8). MS neurons had the shortest RI (0.55 ± 0.052, *N *= 22). Other types of Aβ LTM neurons had an intermediate RI (1.25 ± 0.132 ms, *N *= 18). In neuropathic animals, under the same stimulation conditions, it was found that RI in all Aβ neurons was significantly greater compared with neurons in control animals. RI in MS neurons in neuropathic rats was 1.14 ± 0.17 ms, *N *= 28 (*P *= 0.0038). RI in hair neurons was 2.15 ± 0.152 ms N = 29 (*P *= 0.0002). Aβ HTM neurons also showed significantly greater RI 6.93 ± 1.045 ms, N = 15 (*P *= 0.0171).

Figure 7B (a-b) shows representative examples of MS neuron responses in control and neuropathic rats in response to paired-pulse stimuli. Figure 7B (c-d) shows examples of responses observed in MS neurons in neuropathic rats. In these cases, MS neurons not only showed a greater refractory interval between two APs, but also showed a delayed second AP response to the second stimulus. This delayed response disappeared when the inter-stimulus interval was increased (Figure 7Be).

**Figure 8:**
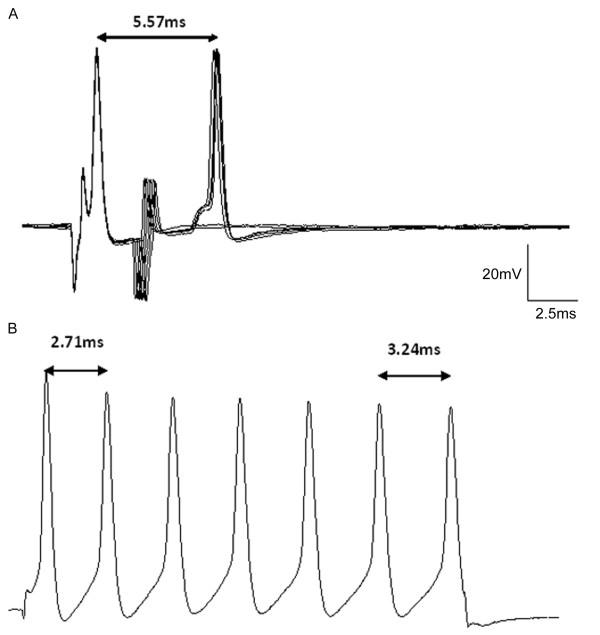
**Comparison of APs evoked by dorsal root paired-pulse stimulation and by soma current injection**. The figure shows APs in one specific MS in a neuropathic animal evoked by dorsal root paired pulse stimulation and by soma current injection. **A**. shows a delayed second response by 2.6~ 2.9 ms at intervals of paired dorsal root stimulation and a failed response at 2.5, 2.6 ms. The actual delayed response interval was at least 5.57 ms. **B**. shows 7 APs in response to depolarizing current injection, with individual AP intervals of 2.71~3.24 ms.

Figure 8 shows APs recorded in one MS neuron in a neuropathic rat evoked by soma current injection and by dorsal root paired-pulse stimulation, to compare the minimum inter-AP intervals evoked by each of these two types of activation. Figure 8A shows an inter-AP interval of 2.6~ 2.8 ms in response to paired-pulse dorsal root stimulation. The actual delayed response interval was at least 3.3 ms. Figure 8B shows 7 responses with individual inter-AP intervals of 2.7~2.9 ms by direct soma stimulation by injection of depolarizing current. For this MS neuron in a neuropathic rat the minimum inter-AP interval in response to intracellular injection stimulation was 2.71 ms while that in response to paired-pulse stimulation of the dorsal root was 5.57 ms. This implies that the axon exhibited a decreased excitability compared to the soma. Further, although this neuron exhibited a delayed response to paired-pulse stimulation it showed 7 APs in response to the intracellular injection of current, which is higher than the average of response in neuropathic animals (5.44 ± 0.440 ms). This implies an attenuated excitability of the axon that did not affect the enhanced excitability in the soma. Thus, we can conclude that the ectopic discharge characteristics of this neuropathic model are an increased excitability of DRG but an attenuated decreased excitability of the dorsal root axon.

## Discussion

### General characteristics of neuropathic afferent neurons

Sensitization is often considered to play a major role in the neural basis of hyperalgesia and allodynia associated with peripheral neuropathy. The present study determined the excitability and patterns of discharge in functionally defined DRG neurons, including electrophysiological properties under different conditions of neuronal activation. In addition, some neurons were provoked to display a discharge, including otherwise quiescent neurons. A systematic correlation of neuronal type with expression of discharge indicated that Ab neurons, including LTM and HTM neurons, exhibited increased activity in neuropathic rats, whereas these neurons did not display such activity in control rats. This principle conclusion is consistent with our previous studies on this model [21-23].

These data are also consistent with previous reports on neuropathic rat models. For example, after peripheral nerve injury both axotomized as well as intact afferent neurons supplying skeletal muscle but not skin afferents generate ongoing activity within the DRG [28]. It has also been shown that after nerve injury sensitization of primary sensory neurons is characterized not only by abnormal discharges but also by having a lowered activation threshold and by exhibiting exaggerated responses to various stimuli [8,29-34]. Here we report several signs of altered excitability, specifically of Ab DRG neurons. For example, persisting activity was generated specifically in both Aβ HTM and Aβ LTM neurons. Further, a reduction was also observed in the mechanical response threshold of some subtypes of Aβ-fiber neuron in the neuropathic rats when the distribution of mechanical thresholds receptive fields of the individual neurons was compared. This suggests a change in excitability of peripheral terminal receptors. Intracellular current injection into the soma of Ab DRG neurons generated significantly more APs in neuropathic rats than in controls, which suggests that there is a change in excitability of Ab DRG neuron somata as well.

While the difference in the soma AP response in Ab DRG neurons between control and neuropathic rats suggests that a constitutively enhanced hyperexcitability occurs in these neurons, it is important to point out that in contrast there was an increased current threshold for activation of dorsal roots as well as a delay in the latency of the second AP in response to paired-pulse dorsal root stimulation at shorter inter-pulse intervals. This suggests an attenuated excitability of Aβ axons in the dorsal root. This appears to be a novel finding. To our knowledge previous studies have not compared changes in excitability in soma, axon and peripheral receptor in this or in other animal models of chronic pain.

### Possible mechanisms underlying changes in excitability in different parts of Aβ DRG neurons

The cellular mechanisms underlying generation of abnormal discharge and the changes in activation threshold of the soma and peripheral sensory receptor in Aβ DRG neurons are not clear. It is generally presumed that altered discharge results from the classical Hodgkin-Huxley repetitive firing processes. The ability of theses neurons to sustain repetitive discharge depends on all three major neuronal ion channels, Na^+^, Ca^2+ ^and K^+^. Alterations in the level of expression, cellular localization, distribution and activation kinetics of each of these channel types might be involved in determination of these changes in excitability. Thus, they may have contributed to the difference in excitability between soma, axon and peripheral receptor.

It is also worth considering how injury to the axon may lead to changes in excitability in the somata and the peripheral endings. While no clear answers immediately come to mind, some studies on models of peripheral neuropathy might offer some insight. For example, Walters and Ambron [35] hypothesized that axonal injury might unmask nuclear localization signals in certain axoplasm proteins at the injured site. It was suggested that this may cause the transport of cytokines, nerve growth (NGF) or glial cell line-derived neurotrophic factor (GDNF), tumor necrosis factor (TNF) or other inflammatory mediators released by immune cells and Schwann cells [36-42], to be transported retrogradely to the somata and activate transcription factors, which then induce the alternation in expression of neuropeptides, receptors and ion channels in the somata. This in turn may induce increased excitability in the DRG somata. In fact, a previous study provided evidence that DRG somata exhibit *de novo *expression of TTX-sensitive type III Na^+ ^channels, a reduction of TTX-resistant Na^+ ^current, and a change in K^+ ^and Ca^+ ^currents in different neuropathic animal models [31,43-48].

The mechanisms underlying the changes in the activation threshold of the peripheral terminals might be related also to alterations in the release of cytokines and growth factors. Previous studies have shown that TNF-α is unregulated during Wallerian degeneration [39] and that exogenous administration of TNF-α produces a direct activation and sensitization of injured afferent fibers [49]. When exposed to NGF sensory neurons undergo an increase in either the firing of APs evoked by a ramp of the depolarizing current [50,51] or in currents evoked by the application of capsaicin [52].

There is no clear explanation for the changes in excitability of the MS neurons. Muscle spindles are the sensory endings primarily responsible for initiation of proprioception. These receptors can be exquisitely sensitive, responding to light tapping, vibration or pressure applied to the skin overlying the muscle spindle within the muscle belly [53]. However, unless there are adequate changes in muscle length, the muscle spindle will not be excited. In the present study we observed a significantly decreased mechanical threshold of MS neurons. However, the mechanisms underlying this change in excitability remain to be determined.

Although there was no direct evidence to explain the attenuated excitability of dorsal root axons in response to direct electrical stimulation, previous studies have shown that the behavioral and cellular effects of nerve injury to the central branches, such as partial dorsal rhizotomy, are different from those induced by injury to the peripheral branches, such as chronic constriction injury of the sciatic nerve. For example, Sheen et al. failed to observe a neuropathic pain behavior after a dorsal rhizotomy [54], while Song et al. found that partial dorsal rhizotomy produced significantly less severe hyperalgesia [13]. These studies suggest that the dorsal root might generate different injury signals and might transport such signals to the spinal cord rather than to DRG somata [13]. Based on our results, we further suggest that the attenuated excitability of dorsal root axons might act as a "negative injury signal", while the increased excitability of somata and peripheral receptors might act as a "positive injury signal" to the central nervous system in this model of peripheral neuropathic pain. Future studies are necessary to test this suggestion.

### Consequences of activity of Aβ neurons

In this model, a significant correlation was found between several variables of discharge and pain behaviors. The "positive" correlation along with the "negative" correlation, as shown in this study, suggest that altered excitability may be important for maintaining neuropathic pain behavior.

Although not each nerve injury necessarily leads to pain, our previous studies in this model have shown that light tactile stimulation induces brisk paw withdrawal [55,56]. Further, innocuous touch stimulation of the cutaneous receptive field induces an excessively prolonged afterdischarge in spinal wide dynamic range neurons and this unusually prolonged afterdischarge of dorsal horn neurons is reduced by peripheral nerve block [22,23]. This present study has shown that Ab DRG neurons exhibit increased discharge and excitability, which includes a prolonged discharge firing pattern in response to direct soma excitation and receptive field stimulation. Thus, it seems reasonable to suggest that the prolonged nature of the afterdischarge in Ab neurons in this model of peripheral neuropathy may lead to greater peripheral drive and that mechanical allodynia, and perhaps also the burning pain, associated with peripheral neuropathy may be due to this increased activity in Ab neurons delivering excitation to nociceptive neurons in the spinal cord.

Thus, based on previous evidence from other laboratories as well as our findings on functionally-identified DRG neurons in this intact nerve model of peripheral neuropathy, we propose that the hyperalgesia and dysesthesia experienced by people with peripheral neuropathy might be at least partially due to sustained discharge of Ab primary afferents in response to light tactile or noxious stimulation of the skin. We further postulate that the altered excitability of Ab neurons that develops in neuropathic rat models, as cited above, may be a critical factor in signaling mechanical allodynia and may contribute to allodynia and spontaneous pain following peripheral nerve injury in humans.

Overall, our data provide specific evidence for a major contribution of altered excitability of Ab neurons deriving from multiple regions of these neurons, including the receptive field and the somata, possibly modified by altered excitability of dorsal root axons, resulting in altered spinal nociceptive mechanisms in this animal model of painful peripheral neuropathy.

## Conclusions

This study has demonstrated changes in excitability of Ab HTM and LTM primary sensory neurons in the nerve-intact cuff model of peripheral neuropathic pain and that different sites of these sensory neurons exhibit different changes in excitability after sciatic nerve injury. These differences consisted of a reduced mechanical activation threshold and abnormal prolonged discharges following receptive field stimulation, a reduced current activation threshold of the cell body and a greater number of APs evoked by intracellular current injection into the soma, yet an increased current activation threshold and delayed recovery kinetics in dorsal root axons. This study provides new evidence to support a role for injury-induced plasticity of primary sensory neurons in this model of neuropathic pain and also indicates that changes to different regions of the sensory neurons might have different effects on peripheral drive to central mechanisms and thus possibly also hyperalgesia or allodynia.

## Methods

All experimental procedures were approved by the McMaster University Animal Research Ethics Board and animals were treated in accordance with the Guide to the Care and Use of Experimental Animals, Vols. 1 and 2, of the Canadian Council on Animal Care.

### Experimental animals and model induction

Young male Sprague-Dawley rats (obtained from Charles River Inc. St. Constant, QC, Canada) weighing 170-200 g were used. Animals were divided into two groups, control and neuropathic model groups. A peripheral neuropathy was induced according to the method previously described in detail [20,57]. Briefly, under anesthesia with a mixture of ketamine (Ketamine; 5 mg/100 g; Bimeda-MTC Animal Health Inc.; Cambridge, ON, Canada), xylazine (Rompun; 0.5 mg/100 g; Bayer HealthCare, Toronto, ON, Canada) and acepromazine (Atravet; 0.1 mg/100 g; Ayerst Veterinary Laboratories, Guelph, ON, Canada) given i.p., the right sciatic nerve was exposed at the mid-thigh level. Two cuffs of 0.5 mm polyethylene tubing (Intramedic PE-90, Fisher Scientific Ltd., Whitby, Ontario, Canada) were inserted around the exposed nerve and placed approximately 1 mm apart. The incision was then sutured in two layers, muscle and skin, and the animals allowed to recover from anesthesia. Antibiotic ointment (Furacin; nitrofurazone 0.2%; Vetoquinol N.-A. Inc.; Lavaltrie, QC, Canada) was applied over the sutured incision and 0.01 ml/100 g of antibacterial injectable solution (Bayer HealthCare, Toronto, ON, Canada) was injected subcutaneously (s.c.). Animals were given 1 ml saline s.c., and ocular lubricant, and placed under a heating lamp until they recovered from the anesthetic. They were then returned to their home cages.

### von Frey test of paw withdrawal threshold

In all cases, the von Frey test was run on the same day as the recording before the rats were anesthetized for the acute electrophysiological experiment; this was to confirm the establishment of a behavioral tactile hypersensitivity, a hallmark of this model of peripheral neuropathic pain. To quantify mechanical sensitivity of the foot, brisk foot withdrawal in response to application of von Frey filaments was measured as described previously [58]. The rats were placed in a transparent Plexiglas box with a clear Plexiglas floor, containing 0.5 cm diameter holes spaced 1.5 cm apart to allowed full access to the paws [22]. Each rat was allowed to habituate to the box for approximately 15 min, until cage exploration and major grooming activities had ceased.

Calibrated von Frey filaments (Stoelting Co., Wood Dale, IL, USA) were applied to the plantar surface of the hind paw to determine the withdrawal threshold. Each von Frey filament was applied 5 times (for 3-4 sec each, at 3 sec intervals) to a different spot on each hind paw. These filaments were applied in ascending order, beginning with the finest filament, until a clear withdrawal response was observed. When this occurred, the next lightest filament was applied following the same procedure. A 50% withdrawal response threshold was derived according to responses to this testing regimen [59] using the up-down method of Dixon [60]. A brisk foot withdrawal in response to these innocuous mechanical stimuli was considered the mechanical threshold.

### Intracellular recording in vivo

The acute electrophysiological experiment was run during the third week after induction of the model. Approaches to the animal preparation and intracellular recording techniques have been reported previously [61,62]. In brief, each animal was initially anesthetized with ketamine mixture described above. The right jugular vein was catheterized for i.v. infusion of drugs and a cannula was inserted into the trachea. The rat was then fixed in a stereotaxic frame and the vertebral column rigidly clamped at the L2 and L6 vertebrae. The right femur was fixed by a customized clamp onto the stereotaxic frame to minimize movement of the DRG during mechanical searching for receptive fields on the leg. The L4 DRG was selected for study as it contains one of the largest numbers of the hind leg afferent somata. A laminectomy was performed to expose the ipsilateral L4 DRG. The L4 dorsal root was sectioned close to the spinal cord, allowing a 12-15 mm length, and the proximal end was placed on a bipolar electrode (FHC, Bowdoinham, ME, USA) used for stimulation purposes. The exposed spinal cord and DRG were covered with warm paraffin oil at 37°C to prevent drying.

The rat was mechanically ventilated via the tracheal cannula using a Harvard Ventilator (Model 683, Harvard apparatus, Quebec, Canada). The ventilation parameters were adjusted so that end-tidal CO_2 _concentration was maintained around 40-50 mmHg, as measured using a CapStar-100 End-Tidal CO_2 _analyzer (CWE, Ardmore, PA, USA). Rectal temperature was maintained at ~37°C using a temperature controlled infrared heating lamp. Immediately before the start of recording, the animal was given 20 mg/kg of Na pentobarbital (CEVA SANTE ANIMAL, Libourne, France), and supplemental doses of 10 mg/kg of pentobarbital were given each hour through the jugular catheter to maintain a surgical level of anesthesia. In addition, just before recording, each animal was also given an initial 1 mg/kg dose of pancuronium bromide (Pavulon, Sandoz, Boucherville, QC, Canada) to eliminate muscle tone. Supplemental doses of 1/3 the initial dose of pentobarbital and pancuronium were given about each hour via the jugular catheter.

Intracellular recordings from somata in the exposed DRG were made with borosilicate glass micropipettes (1.2 mm outside diameter, 0.68 mm inside diameter; Harvard Apparatus, Holliston MA, USA). The electrodes were pulled using a Brown-Flaming puller (model p-87; Sutter Instrument CO., Novota, CA, USA) and were filled with 3 M KCl (DC resistance 50-70 MΩ). Signals were recorded with a Multiclamp 700B amplifier (Molecular Devices, Union City CA, USA) and digitized on-line via Digidata 1322A interface (Molecular Devices, USA) with pClamp 9.2 software (Molecular Devices, USA). The microelectrode was advanced using an EXFO IW-800 micromanipulator (EXFO, Montreal, QC, Canada) in 2 μm steps until a hyperpolarization of at least 40 mV suddenly appeared. For any testing to proceed a continuous recording was obtained for at least five minutes after cell penetration; stable recordings were obtained for periods exceeding one hour. For each neuron, once a stable membrane potential had been confirmed a single stimulus was applied to the dorsal roots to provoke an AP; with the aid of the protocol editor function in pClamp 9.2 software, a somatic AP was evoked by stimulation with a single rectangular voltage pulse.

### DRG neuron classification

DRG sensory neurons were classified during intracellular *in vivo *electrophysiological experiments according to parameters reported previously by other laboratories to distinguish DRG neuron types, including: 1) the configuration of the AP, 2) the conduction velocity, and 3) the response properties to application of natural stimuli to peripheral receptive fields [26,27,61-64].

The sensory receptive properties of each DRG neuron were examined using hand-held mechanical stimulators and classified as previously described. The threshold of activation, the depth of the receptive field and the pattern of adaption were the major factors to further classify neurons into low threshold mechanoreceptor (LTM), high threshold mechanoreceptor (HTM) and unresponsive neurons. LTM neurons were further classified using soft brush, light pressure with a blunt object, light tap and vibration. Many LTM neurons are cutaneous, and include guard/field neurons (GF), rapidly adapting (RA) neurons, Pacinian afferents, slowly adapting (SA) neurons. A group of neurons with deeper receptive fields that were very sensitive to light pressure and/or leg movement and often showed ongoing activity, were classified as muscle spindle (MS) neurons. These neurons also exhibited slow adaptation to dorsal root stimulation, to intracellular injection of depolarizing current and to leg movement. HTM neurons responded to noxious stimuli including noxious pinch and application of sharp objects such as the end of a syringe needle. Neurons that did not respond to any of the innocuous or noxious mechanical stimuli listed above were classed as unresponsive [26]. Heat nociceptors and specific cooling receptors were not included in this study due to the very low numbers of such neurons.

Neurons were also classified according to dorsal root CVs: C-fiber neurons (≤ 0.8 mm/ms), Aδ-fiber neurons (1.5-6.5 mm/ms) and Aα/β-fiber neurons (> 6.5 mm/ms). This classification has been used as a means of classification of neurons in other models of peripheral neuropathy [7,10,13,65,66].

Compared to other criteria from other groups [64,67], these criteria most closely matched the present study, including similar surgical procedure, recording technique and setting, etc. It should be noted that as excitability of sensory neurons can be altered in models of peripheral neuropathy, functional classification was based primarily on responses to activation of the peripheral receptive fields. However, classification was also based on AP configuration and on responses to activation.

### Stimulation from different sites of sensory neurons

The various parts of the primary afferents were stimulated to determine excitability measured as evoked APs in the soma, including stimulation of the soma by direct depolarizing current injection, electrical stimulation of the dorsal roots using bipolar stimulating electrodes and activation of the peripheral receptive field.

Soma-To quantify soma excitability, with the aid of the protocol editor function in pClamp 9.2 software (Molecular Devices) the threshold of depolarizing current pulses injected into the soma was determined, applying current pulses of 100 ms in increments of 0.05 nA through the recording electrode until an AP was elicited or until a maximum current of 4 nA was reached. The excitability of the soma was also evaluated by comparing the number of APs evoked by injecting defined current pulses to the DRG soma; three intracellular current injections of 20 ms each were delivered with 1, 1.5 and 2 nA.

Dorsal roots-Dorsal root excitability was measured by determining the chronaxie curve (threshold-duration), which was defined by delivering the minimum current that would elicit an AP in the soma to the dorsal root using current pulse durations of 0.1, 1, 2, 4 and 6 ms. The stimulation pulse was delivered from an S940/910 stimulus adaptor/isolator (Dagan, Minneapolis, MN, USA). Dorsal root excitability was also tested by measuring the refractory interval of two APs to paired-pulse stimulation. Stimulation started with search stimuli with coarse interval steps down of 1 ms, followed by final interval steps at 0.1 ms. Stimulus pairs had intervals of 1-80 ms and this interval was decreased gradually at final interval steps with a pause of 4 s between pairs of stimuli. For each neuron, the recording with the smallest RI (refractory interval) was chosen for analysis.

Peripheral receptive field-The mechanical sensitivity of DRG neurons was determined individually with calibrated von Frey filaments as described by other groups [26,64]. After classification of each DRG neuron, the size and localization of the receptive field using von Frey filaments applied to the receptive field were determined. Mechanical threshold of these neurons was determined as the minimum force, in g, necessary to evoke APs. In most cases von Frey filaments were applied to the most sensitive spot on the skin. For Aβ G-hair and Aδ D-hair neurons the von Frey filaments were applied to the tips of the hairs, while for Aβ field-hair neurons the von Frey filaments were applied the skin next to the base of the hairs. For MS neuron, von Frey filaments were applied the skin along the region the muscle belly and the joint. Neurons that were not responsive to von Frey filaments were excluded in this part of the study. The mechanical stimuli forces exerted with the calibrated von Frey filaments used in this study were a set of von Frey filaments (0.008, 0.02, 0.04,0.07, 0.16, 0.4, 0.6, 1.0, 1.4, 2.0, 4.0, 6.0, 8.0, 10, 15, 26, 60, 100, 180 and 300 g; 1.65-6.65 mm tip-diameters).

### Statistical analysis

The data are represented as means ± SEMs. An unpaired t-test was used for comparison of the response properties of the neurons between control and neuropathic animals unless otherwise stated. All statistical tests and graphing were done using Prism4 software (Graphpad, La Jolla, CA, USA). P-value is indicated in the graphs and P < 0.05 was considered to indicate a statistically significant difference.

## Authors' contributions

JLH conceived of, designed, and coordinated the study. YZ did the electrophysiological experiments, analyzed the data and performed statistical analyses. YZ wrote the initial draft of the manuscript. Both authors worked on refining this draft and the revision based on editorial review. Both authors have read and approved the final manuscript.
